# DRAM-3 modulates autophagy and promotes cell survival in the absence of glucose

**DOI:** 10.1038/cdd.2017.57

**Published:** 2017-06-23

**Authors:** M Mrschtik, J O'Prey, L Y Lao, J S Long, F Beaumatin, D Strachan, M O'Prey, J Skommer, K M Ryan

**Correction to:** *Cell Death and Differentiation* (2015) **22**, 1714–1726; doi:10.1038/cdd.2015.26; published online 1 May 2015

This article was originally published under NPG’s License to Publish, but has now been made available under a CC BY 4.0 license. The PDF and HTML versions of the paper have been modified accordingly.

